# Papillary renal cell carcinoma in an ectopic intrathoracic kidney within Bochdalek hernia: A case report

**DOI:** 10.1016/j.eucr.2025.103217

**Published:** 2025-09-16

**Authors:** Erin Catherine Schnell, Madeline Elise Becker, Paul Gerard McQuillen, Alexandra Paige Foster, Elizabeth Ann Allen, Jeffrey Edward Bransky, Joshua Zachary Nellis, Joseph Michael Zavell, James N. Conner, Jeffrey K. Mullins

**Affiliations:** University of South Carolina- Prisma Health Department of Surgery, CHI Memorial- Department of Urology, USA

## Abstract

Intrathoracic kidneys, an uncommon form of ectopic kidneys, often occur with congenital diaphragmatic hernias. We report a case of a 72-year-old male with a left-sided Bochdalek hernia containing an intrathoracic kidney, incidentally discovered on imaging for abdominal discomfort and respiratory symptoms. Imaging revealed a suspicious renal mass, which led to a partial nephrectomy and the diagnosis of papillary renal cell carcinoma (pRCC), a rare finding in ectopic kidneys. Postoperative follow-up demonstrated no recurrence or metastasis. This case highlights the importance of monitoring intrathoracic kidneys for potential complications and the challenges in diagnosing and managing ectopically located renal tumors.

## 1 Background

An intrathoracic kidney is a very rare form of ectopic kidney that often occurs in individuals with a left-sided posterior congenital diaphragmatic hernia. Intrathoracic ectopic kidney accounts for less than 5% of all renal ectopias, with a reported incidence of 0.25 %.^1^^,^^2^ Although rarely cited, intrathoracic kidneys have been classified into four subtypes. These include (a) closed diaphragm, (b) eventration of the diaphragm, (c) diaphragmatic hernia (congenital or acquired) and (d) traumatic rupture of the diaphragm.^1^ The first two are usually asymptomatic.^1^ As discussed in this case, ectopic kidney associated with congenital diaphragmatic defects such as Bochdalek hernia (subtype c) is a rare clinical phenomenon, with a reported incidence of 0.25 %.^3^

A Bochdalek hernia is a congenital defect in the posterolateral diaphragm caused by incomplete maturation of the pleuroperitoneal folds. This defect allows abdominal viscera to herniate into the thoracic cavity. Bochdalek hernias more often occur on the left side (70–90 % of cases), presumably due to the fact that the pleuroperitoneal canal closes earlier on the right side.^4^^,^^5^ During embryogenesis, the kidneys develop in the pelvis and normally ascend to T12-L3 between the sixth and ninth weeks.^6^ Diaphragmatic defects can disrupt this ascent.^5^

The contents of the Bochdalek hernia vary. Large and small bowel and stomach are most frequently involved, but very rarely contain the kidney.^4^ Clinical presentation varies with timing. When occurring in neonates, Bochdalek hernias may present with significant risks, including possible pulmonary hypoplasia or persistent fetal circulation.^7^ Delayed presentation in adults is usually asymptomatic and is often discovered incidentally on imaging.^7^ In adults, there is male predominance (3:1) and left-sided predominance.^4^

In most individuals, an intrathoracic kidney is benign and asymptomatic.^5^ When symptomatic, presentations may include kidney stones, pyelonephritis, hydronephrosis, or respiratory distress from lung compression.^3^ Given the variable symptoms, diagnosis is often incidental. CT and magnetic resonance imaging (MRI) are preferred imaging tests for detecting ectopic kidneys.^5^ In such asymptomatic patients, treatment is not required.^8^ Intervention is only required for complications like malignancy.^5^ Surgical guidelines are not well defined.^3^ Histologically, pRCC is differentiated by the presence of tumor cells in a papillary architecture.^8^ Metastatic dissemination of pRCC commonly involves lungs, bone, liver, brain, and lymph nodes._8_ However, compared to clear cell RCC, pRCC tumors are more likely to be localized at the time of diagnosis._8_ Clinically, pRCC presents variably, with hematuria, weakness, and flank pain.^9^ As pRCC tumors are usually hypovascular, diagnostic imaging such as multi-phase renal imaging can differentiate pRCC.^8^ MRI shows sensitivity of 85.6 % and specificity of 91.7 %._8_

RCC occurring in an ectopic kidney is an extremely rare clinical event.^10^ Literature review shows very few cases with variable presentations.^9^ Due to the many unusual presentations of RCC, missed or delayed diagnoses are common and increase morbidity and mortality. To date, only one prior case of RCC in an intrathoracic kidney has been published.^10^ We report a 72-year-old male with papillary RCC (pRCC) in an intrathoracic kidney within a Bochdalek hernia.

## 2 Case

A 72-year-old male with hypertension, coronary artery disease, thyroid disease, osteoarthritis, COPD, and a known intrathoracic left kidney within a chronic Bochdalek hernia presented to clinic with dull, intermittent upper abdominal pain, shortness of breath, and several months of progressive dyspnea, requiring home oxygen therapy. He denied urinary symptoms. This patient previously presented to his primary care physician three months prior with a similar complaint of shortness of breath and workup with computed tomography (CT) of the chest was performed. At that time, CT with IV contrast of the chest showed stable large left posterior diaphragmatic hernia containing mesenteric fat and the left kidney consistent with a Bochdalek hernia, which was unchanged from prior imaging of his intrathoracic kidney in 2001. There were no evident signs of active pulmonary disease on imaging. Of note, this patient has a remote history of urolithiasis and known renal cysts and is status post left hydrocelectomy in 2001. He quit smoking 25 years ago.

**Fig. 1:**
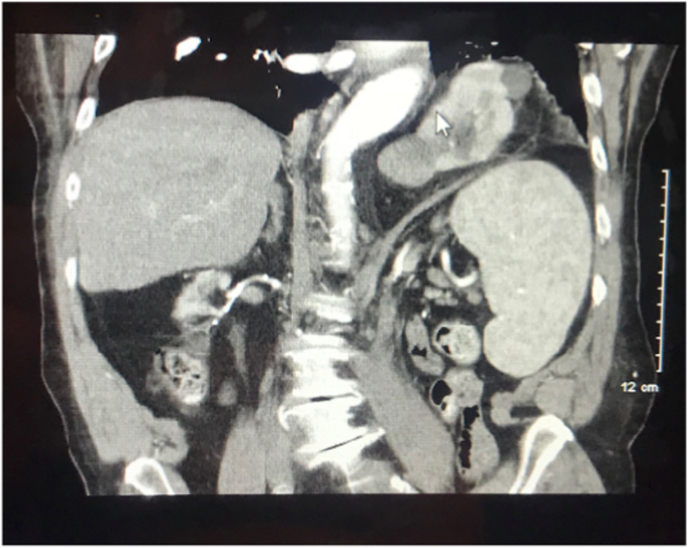
CT of the abdomen shows an intrathoracic left kidney within left diaphragmatic hernia and an intermediate density lesion arising from the anterior mid left kidney measuring 3.9 × 3.6 cm.

A follow-up CT of the abdomen was ordered and showed consistent anatomy with prior confirmed intrathoracic left kidney within left diaphragmatic hernia. Repeat CT imaging additionally highlighted an intermediate-density lesion arising from the anterior mid-left kidney measuring 3.9 × 3.6 cm (Fig. 1). To further categorize this density out of concern for malignancy, additional imaging with four-phase renal CT was obtained. On four-phase renal CT, the left intermediate-density lesion showed a pre-contrast attenuation of 33 Hounsfield units and post-contrast of 46–75 Hounsfield units, thus concerning for malignancy.

The patient was referred to a urologic oncologist to discuss imaging results and possible treatment options. A preoperative diagnosis of a cT1a left renal mass in the intrathoracic kidney was made. The nature of the renal mass and likelihood of malignancy was discussed with the patient, as well as possible treatment options including active surveillance, biopsy, ablation, partial nephrectomy, and radical nephrectomy. Biopsy or ablation raised concern for complications such as risk of progression, possible metastases, inaccurate biopsy, and incomplete ablation**.** With the four-phase renal CT suggestive of malignancy, a radical or partial nephrectomy presented the best options to mitigate risk with the patient opting for the latter. A thoracic surgeon was consulted, but advised against repair because the patient did not have enough pulmonary reserve. As such, the following risks of not repairing the hernia require continued monitoring for respiratory compromise, herniation of abdominal contents, and strangulation of other organs. The patient consented for and underwent a robotic-assisted left partial nephrectomy of the intrathoracic kidney with biopsy. Partial nephrectomy was chosen based on research supporting that it is the gold standard for renal preservation. The patient was placed in reverse Trendelenburg, four robotic ports were placed, and transperitoneal entry was achieved. The port sites chosen were the standard sites for partial nephrectomy; a camera port below the costal margin on the ipsilateral pararectal line, two working ports along the same line, and an assistant port in the lower quadrant. Intraoperatively, a biopsy of a suspicious node was taken. The resected specimen was extracted laparoscopically.^11^

Pathology of the resected intrathoracic kidney specimen revealed a grade 1 pRCC measuring approximately 4.8 × 4.3 × 3.2 cm, which was larger than the lesion dimensions on preoperative CT imaging (3.9 x 3.6 cm). Margins were negative. Pathology of the nodal biopsy was negative, indicating no spread to the lymph nodes. Following surgery, the patient recovered well, and the remainder of his postoperative course was uneventful. He was ultimately discharged home and was scheduled to follow up with his surgeon in 6 months. At his 6-month follow-up, he had repeat imaging done, including CT of the abdomen and pelvis and chest radiography. Subsequent imaging showed no evidence of recurrence and ruled out typical sites of pRCC metastasis including lymph nodes, lungs, liver, and bones. The tumor was staged as pT1aN0M0 pRCC, given no nodal involvement and no sites of metastasis.

## 3 Discussion

In this case report, we discussed an unusual presentation of a pT1aN0M0, grade 1 pRCC in an intrathoracic kidney. This patient presented with many risk factors for pRCC including history of hypertension, male sex, previous smoker, and remote history of renal cysts and urolithiasis. To our knowledge, there is one previous case report of RCC occurring in an intrathoracic ectopic kidney, illustrating the rare occurrence of renal tumors associated with ectopic kidneys._10_

As in this case, presentation of pRCC in an intrathoracic kidney associated with Bochdalek hernia in adulthood is unusual, and thus difficult to diagnose. Clinically, the patient presented with shortness of breath and abdominal pain, lacking typical symptoms seen with pRCC including hematuria, weakness, and flank pain.^9^ In the setting of a left Bochdalek hernia and involvement of kidney in the hernia, the patient's shortness of breath and abdominal pain could be secondary to lung compression and herniation of abdominal viscera respectively.^4^ Additional possible complications associated with intrathoracic kidneys in the setting of Bochdalek hernias include kidney stones, pyelonephritis, and hydronephrosis.^3^ Of note, in this case, he also had a history of kidney stones. Though remarkably rare, malignancy affecting the thoracic kidney is a potential clinical complication presented in this case.^8^

Due to asymptomatic presentation and rarity of this condition, the treatment guidelines for RCC in intrathoracic kidneys are not well defined. Localized pRCC can be managed with partial or radical nephrectomy, ablation, or active surveillance.^8^ Surveillance, biopsy, or ablation risk progression or incomplete treatment.^8^ As performed in this case, partial nephrectomy is the standard treatment for localized tumors in ectopic kidneys.^3^ Past cohort studies have shown excellent recurrence-free survival rates with partial nephrectomy, with one cohort study showing 5- and 10-year RFS of 95.8 % and 73 %, respectively in patients with pT1a pRCC who underwent partial nephrectomy.^8^ Partial nephrectomy additionally serves as the gold standard for treatment given renal preservation and potential to minimize long-term kidney disease.^8^ A surgical treatment approach can vary depending on the location of tumor, clinical stage of malignancy, and comorbid conditions. Other surgical considerations include the age of the patient, additional organs located within the hernia, and proximity of vasculature. This case successfully used a robotic laparoscopic approach through the abdomen without necessitating additional specialties intraoperatively. A thoracic surgeon was consulted for decision-making regarding the diaphragmatic hernia repair.

In infants, or when major organs or vasculature are involved, multidisciplinary management may be required. Prior examples of surgical management for an intrathoracic kidney with chronic diaphragmatic hernia have used a combined thoracoscopic and laparoscopic techniques. In these prior cases, the diaphragmatic hernia was repaired using a thoracoscopic approach and the kidney was returned to the abdomen using a laparoscopic approach.^11^ In our case, diaphragmatic repair was not performed given the patient's limited pulmonary reserve among other considerations.

Ectopic kidneys often have complex vasculature, complicating surgery.^3^ For surgical management in somewhat similar cases of RCC in an ectopic pelvic kidney, a multidisciplinary team has been organized to ensure patient safety. This is due to a variety of factors, but most notably, the complex vasculature that can form in the tumor. This seems to be especially true if the ectopic tumor is located near the aorta. As such, in addition to urologists operating as the lead surgeons, radiological services must be involved to map out the complex vasculature. Authors vary on the kinds of radiological imaging needed for each case, but suggestions include a 64 slice CT angiogram or an MRA as an alternative approach.^12^

In cases where the patient may be able to tolerate hernia repairs or it is deemed necessary based on the patient's baseline, a thoracoscopic approach can be used for closure of diaphragmatic hernias associated with intrathoracic kidneys with minimal complications.^3^ This allows for a more complete closure of the diaphragmatic hernia with a reduced chance of reoccurrence. Some risks for this particular surgery must be considered for the safety of the patient. Careful attention to the potential adhesion of the lung bottom and hernia sac should be noted, as failure to address it can lead to air leaks from the bronchi, potentially leading to lobectomy, similar to the concerns for our patient. Another risk that must be considered includes manipulation of ureters, as congenital intrathoracic kidneys may present with sustainably longer ureters. Failure to address the length can lead to ureteral folds postoperatively when the kidneys are placed back into the abdomen.^13^ For advanced pRCC, optimal management remains uncertain; targeted and immunotherapies are under study.^8^

This case highlights the importance of monitoring for potentially morbid complications of ectopic intrathoracic kidneys, such as malignancy. Asymptomatic patients with intrathoracic kidneys require surveillance with ultrasound or CT imaging to assess for development of malignancy, tumor growth, metastatic progression, and the need for further surgical evaluation. While renal dysfunction is a known risk factor for pRCC, the exact relationship between ectopic kidney and malignancy remains unclear. The present and previous noted cases illustrate the importance of regular follow-up observations and imaging in patients with intrathoracic kidneys, monitoring for possible morbid complications including malignancy.

## CRediT authorship contribution statement

**Erin Catherine Schnell:** Writing – review & editing, Writing – original draft. **Madeline Elise Becker:** Writing – review & editing, Writing – original draft. **Paul Gerard McQuillen:** Writing – review & editing. **Alexandra Paige Foster:** Writing – review & editing. **Elizabeth Ann Allen:** Writing – review & editing. **Jeffrey Edward Bransky:** Writing – review & editing. **Joshua Zachary Nellis:** Writing – review & editing. **Joseph Michael Zavell:** Writing – review & editing. **James N. Conner:** Writing – review & editing. **Jeffrey K. Mullins:** Writing – review & editing.

## Conflict of interest statement

The authors declare that there is no conflict of interest regarding the publication of this case report. Conflict of interest may arise when personal or financial relationships could unduly influence the research process or its outcomes. In accordance with ethical standards, the authors affirm that there are no affiliations, financial interests, or personal connections that might bias the content or findings presented herein. This ensures the integrity and objectivity of the research conducted and reported in this work.
